# Diagnostic accuracy of the Xpert MTB/RIF assay for tuberculous pericarditis: A systematic review and meta-analysis

**DOI:** 10.1371/journal.pone.0257220

**Published:** 2021-09-10

**Authors:** Guocan Yu, Fangming Zhong, Yanqin Shen, Hong Zheng

**Affiliations:** 1 Department of Thoracic Surgery, Affiliated Hangzhou Chest Hospital, Zhejiang University School of Medicine, Zhejiang Chinese Medicine and Western Medicine Integrated Hospital, Hangzhou, Zhejiang, China; 2 Zhejiang Tuberculosis Diagnosis and Treatment Center, Affiliated Hangzhou Chest Hospital, Zhejiang University School of Medicine, Zhejiang Chinese Medicine and Western Medicine Integrated Hospital, Hangzhou, Zhejiang, China; The University of Georgia, UNITED STATES

## Abstract

**Objective:**

The purpose of this study was to evaluate the diagnostic efficacy of Xpert MTB/RIF for tuberculous pericarditis (TBP).

**Methods:**

We searched relevant databases for Xpert MTB/RIF for TBP diagnosis until April 2021 and screened eligible studies for study inclusion. We evaluated the effectiveness of Xpert MTB/RIF when the composite reference standard (CRS) and mycobacterial culture were the gold standards, respectively. We performed meta-analyses using a bivariate random-effects model, and when the heterogeneity was obvious, the source of heterogeneity was further discussed.

**Results:**

We included seven independent studies comparing Xpert MTB/RIF with the CRS and six studies comparing it with culture. The pooled sensitivity, specificity, and area under the curve of Xpert MTB/RIF were 65% (95% confidence interval, 59–72%), 99% (97–100%), and 0.99 (0.97–0.99) as compared with the CRS, respectively, and 75% (53–88%), 99% (90–100%), and 0.94 (0.92–0.96) as compared with culture, respectively. There was no significant heterogeneity between studies when CRS was the gold standard, whereas heterogeneity was evident when culture was the gold standard.

**Conclusions:**

The sensitivity of Xpert MTB/RIF for diagnosing TBP was moderate and the specificity was good; thus, Xpert MTB/RIF can be used in the initial diagnosis of TBP.

## 1. Introduction

Tuberculosis (TB) is a major global public health threat to human health [1]. Tuberculosis-related mortality remains high in developing countries, especially among those co-infected with acquired immunodeficiency syndrome (AIDS) and tuberculosis [2]. *Mycobacterium tuberculosis* (MTB) can infect almost every part of the body, but the most common site of infection is the lungs, leading to pulmonary tuberculosis (PTB). Infections occurring outside the lungs are referred to as extrapulmonary tuberculosis (EPTB). Severe types of EPTB lead to increased tuberculosis-related mortality [3]. Tuberculous pericarditis (TBP) is a critical type of EPTB, with the human immune deficiency virus epidemic, the incidence of TBP has progressively increased [4]. TBP is the most common cause of pericarditis in areas with a high incidence of TB [4, 5]. In the absence of prompt and effective treatment, TBP can result in very serious consequences, such as pericardial tamponade, constrictive pericarditis, and even death [6]. TBP has a fatality rate of up to 17–40% at longer than six months [5, 7]. To reduce the poor prognosis of TBP, early diagnosis and treatment are essential. However, the early diagnosis of TBP is still very difficult and is often postponed [6]. The reason for this is that the amount of MTB in pericardial fluid is generally very low, which results in a low positive rate for the commonly used acid fast bacillus (AFB) smear, and MTB culture takes weeks to produce results and thus cannot guide early diagnosis [8]. Other tests, such as pericardial effusion adenosine deaminase, although indirectly helpful in the diagnosis, do not provide a direct microbiological basis [9].

Xpert MTB/RIF uses semi-nested real-time polymerase chain reaction to detect MTB DNA in specimens, with the ability to report MTB and rifampicin resistance results within two hours [10, 11]. Based on the good performance of Xpert MTB/RIF in the diagnosis of TB, the World Health Organization has recommended the test for the early diagnosis of TB since 2010 [12]. Xpert MTB/RIF is also applicable to EPTB, such as lymph node TB, and it has also shown excellent diagnostic efficacy [13]. The application of Xpert MTB/RIF in the diagnosis of TBP has its unique advantages. Xpert MTB/RIF makes it possible to find microbiological evidence of MTB in the early and rapid diagnosis of TBP. However, the accuracy of Xpert MTB/RIF in the diagnosis of TBP was still lacking systematic evaluation, the diagnostic validity of Xpert MTB/RIF for TBP compared to different reference standards is still unclear. We performed this systematic review and meta-analysis to synthesise evidence on the diagnostic accuracy of Xpert MTB/RIF for detection of TBP among people living in endemic areas.

## 2. Methods

### 2.1 Design and registration

This was a systematic review and meta-analysis of a diagnostic test accuracy to synthesise evidence on the diagnostic accuracy of Xpert MTB/RIF for detection of TBP. On the International Platform of Registered systematic Review and Meta-Analysis Protocols (INPLASY), we have registered the protocol with the registration number of INPLASY202060045 [14]. The protocol of this meta-analysis had been published in PLOS ONE [15]. This study was performed following the Preferred Reporting Items for Systematic Reviews and Meta-Analysis for Diagnostic Test Accuracy (PRISMA-DTA) guideline [16].

### 2.2 Information sources

We searched the relevant studies in Embase, PubMed, the Cochrane Library, China National Knowledge Infrastructure (CNKI), and the Wanfang database for researches, which assessing the diagnostic accuracy of Xpert MTB/RIF for TBP up to April 2021. We also explored the references cited in reviews for possible researches.

### 2.3 Search strategy

Guocan Yu and Fangming Zhong conducted the search strategies. We restricted to English and Chinese language in our search process. Guocan Yu did study search using search strategies. Search strategy of PubMed was listed as follows:

#1 “Pericarditis, Tuberculous”[Mesh] OR “Pericarditides, Tuberculous” OR “Tuberculous Pericarditides” OR “Tuberculous Pericarditis”

#2 "Tuberculosis"[Mesh] OR tuberculosis OR Tuberculoses OR “Kochs Disease” OR “Koch’s Disease” OR “Koch Disease” OR “*Mycobacterium tuberculosis* Infection” OR “Infection, *Mycobacterium tuberculosis*” OR “Infections, *Mycobacterium tuberculosis*” OR “*Mycobacterium tuberculosis* Infections”

#3 "Pericardial Effusion"[Mesh] OR “Effusion, Pericardial” OR “Effusions, Pericardial” OR “Pericardial Effusions” OR Hemopericardium OR Chylopericardium OR Chylopericardiums

#4 #2 AND #3

#5 "Extra pulmonary tuberculosis" OR " Extrapulmonary tuberculosis"

#6 #1 OR #4 OR #5

#7 Xpert OR geneXpert

#8 #6 AND #7

The Cochrane Library, Embase, CNKI, and Wanfang databases used the similar search formulae.

### 2.4 Eligibility criteria

#### 2.4.1 Type of studies

Prospective study, retrospective study, case-control study or cross-sectional study, if it had evaluated the accuracy of Xpert MTB/RIF for TBP. We excluded case reports, articles written in languages other than Chinese and English, researches with < 10 specimens, conference reports, and abstracts without full articles.

#### 2.4.2 Participants

Participants living in TB endemic areas using Xpert MTB/RIF to diagnose TBP regardless of sex, age, and geographic locations.

#### 2.4.3 Index tests

We considered Xpert MTB/RIF as index test.

#### 2.4.4 Comparator test

Comparator test (tests other than the reference standard) was not an obligatory criteria (single arm study can be enrolled if participants, intervention, outcomes are satisfied because this study measured the diagnostic accuracy of Xpert MTB/RIF for TBP.

#### 2.4.5 Outcomes

The main outcome was measured in terms of sensitivity and specificity of the index test. Sensitivity refers to the probability that the index test result was positive in an infected case. Specificity refers to the probability that the index test result was negative in a non-infected case [17, 18]. True positive (TP), false positive (FP), false negative (FN), and true negative (TN) values for the index test can be extracted or calculated directly from the studies.

#### 2.4.6 Target conditions

Full-text original researches that assessed the Xpert MTB/RIF assay for TBP were included. TBP was as defined by the authors in the primary studies. Clear and appropriate reference standards were defined in researches.

#### 2.4.7 Reference standards

A composite reference standard (CRS) or MTB culture was defined as the reference standard in our study. Clinical symptoms, radiographic features, biochemical test results, smears, culture, histopathology, and response to anti-tuberculosis drugs constituted the reference standards in the CRS. Some or all of the factors with positive results were considered positive for TBP. Cases were considered as non-TBP if all the results are negative. We used the CRS as defined in the original paper.

### 2.5 Literature screening and selection

Primary search records were imported into ENDNOTE X9.2 literature management software, according to eligibility criteria. Two investigators (Guocan Yu and Fangming Zhong) independently assessed the candidate articles by reviewing their titles and abstracts, followed by the full text, for inclusion. Discrepancies between the two investigators were resolved by discussion with a third investigator (Hong Zheng).

### 2.6 Data extraction

We extracted data including first author name; publication year; country; TP, FP, FN, and TN values for the assay; cut-off value of the index test, reference standard; patient selection method; specimen type; some steps (e.g., homogenization); and condition along with other parameters. The same two investigators (Guocan Yu and Fangming Zhong) independently extracted the necessary information from each of the included articles; We cross-checked the data that we have obtained. Discrepancies in the two data sets were settled by discussion with a third investigator, similar to the literature selection phase. Data from studies against two different reference standards were treated separately.

### 2.7 Quality evaluation

Based on the two reference standards (CRS and culture), the two investigators independently divided the studies into two groups and used a revised tool for Quality Assessment of Diagnostic Accuracy Studies (QUADAS-2) to assess study quality separately [19] and the discrepancy between the two investigators was solved by discussion with a third investigator (Hong Zheng). QUADAS-2 comprises four domains: patient selection, index test, reference standard, and flow and timing. Each domain is assessed in terms of risk of bias, and the first three domains are also assessed in terms of concerns regarding applicability.

### 2.8 Data synthesis and statistical analysis

We first obtained the values corresponding to TP, FP, FN, and TN in each included study, and calculated the estimated pooled sensitivity and specificity of the Xpert MTB/RIF associated with the 95% confidence interval (CI), against CRS or culture, using bivariate random-effects models. Forest plots for sensitivity and specificity were generated for each study. The areas under summary receiver operating characteristic (SROC) curves (AUC) were subsequently calculated. Plots observed data in ROC plane for visual assessment of threshold effect. *I*^2^ statistics was used to assess heterogeneity between the studies and a reference standard. While 0% indicated no observed heterogeneity, values greater than 50% was considered to imply substantial heterogeneity [20]. We explored different types of samples, different patient selection methods, decontamination methods, sample conditions, and homogenization as potential sources of heterogeneity, using subgroup and meta-regression analyses. Sensitivity analyses were used to reanalyses studies without poor quality in terms of QUADAS-2 to check the robustness of analyses. At least four published studies were required to perform the meta-analysis for predefined variable types. Data from studies against CRS and culture were analyzed separately. According to the PRISMA-DTA statement, systematic review and meta-analysis of diagnostic test accuracy studies is not required to assess publication bias [16]. Stata version 15.0 (Stata Corp., College Station, TX, USA) with the *midas* command packages was used to generate forest plots of sensitivity and specificity with 95% CI for each study and carry out meta-analyses and meta-regression analyses. The Grading of Recommendations Assessment, Development and Evaluation (GRADE) guideline was used to assess the strength of the body of evidence [21]. The quality of evidence was classified into 4 levels: high, moderate, low, and very low, and the strength of the recommendation was graded as strong or weak.

## 3. Results

### 3.1 Identification of studies and study characteristics

**Fig 1:**
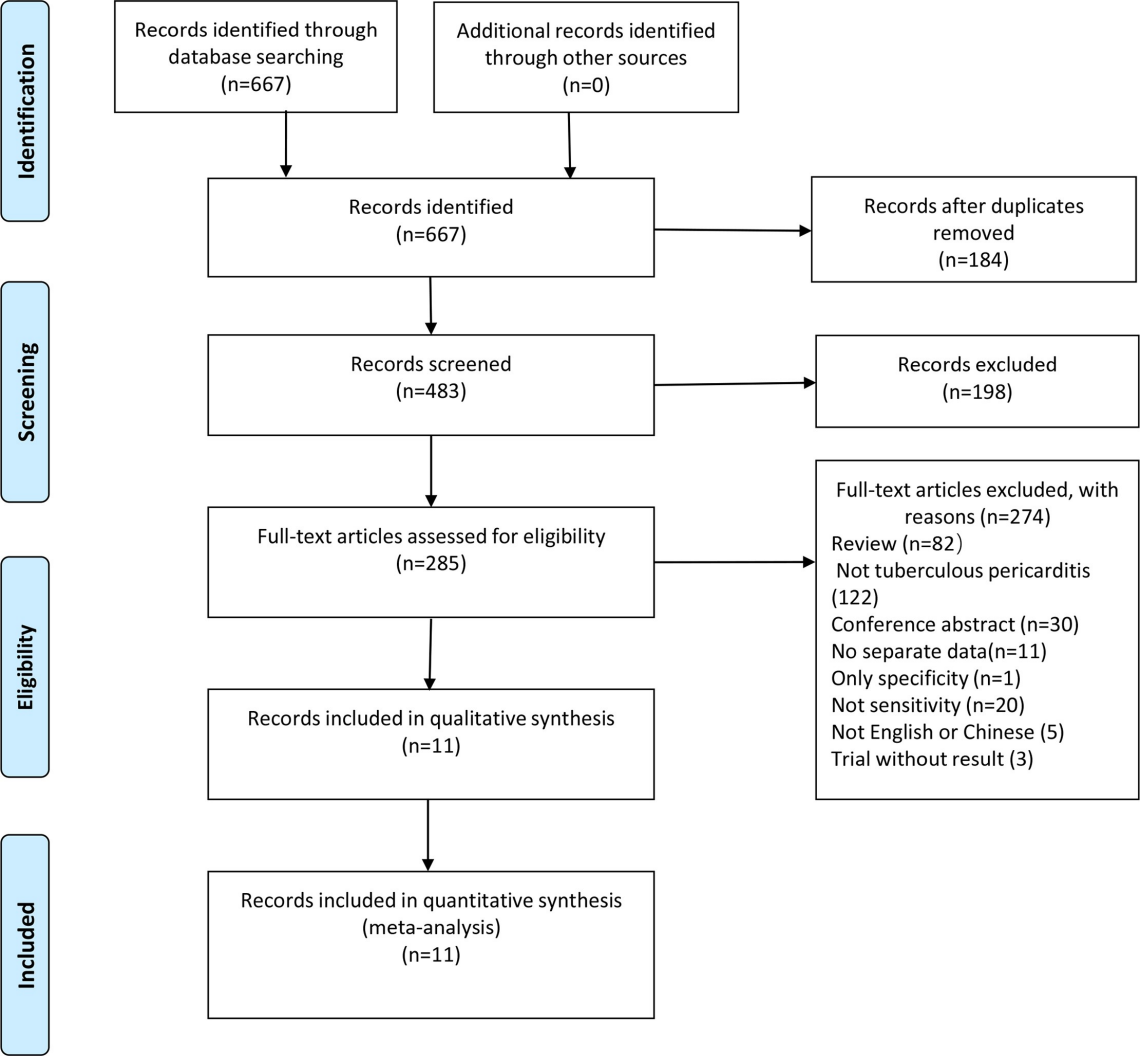
Literature retrieval flow chart. A total of 305, 122, 8, 147, and 83 articles were identified in the Embase, PubMed, the Cochrane Library, China National Knowledge Infrastructure (CNKI), and the Wanfang databases, respectively.

We identified 667 candidate articles from our search of the relevant databases. Based on the inclusion criteria, we screened eleven articles for eligibility (Fig 1) [22–32]. The kappa index of agreement for the selection and data extraction was 0.846 (95% CI, 0.734–0.958) between the two investigators. All of the studies were conducted in low-income areas with high burden of TB. All patient populations were patients with suspected TBP, and Xpert MTB/RIF was used as a screening diagnosis tool. Only one of the studies included in this study reported human immunodeficiency virus (HIV) infection status [26], while the other studies did not report HIV infection status. Two articles were published in Chinese [28, 30] and the remaining nine articles in English. The number of specimens included in the eligible articles ranged from 16–180, and the average specimen size was 83.8. We excluded one other article that reported on the specificity only [33], without reporting any sensitivity. We excluded five articles written in languages other than Chinese or English [34–38]. The specimens used in the article were pericardial effusion, pericardial tissue, or both.

In cases in which the same article reported relevant results for two different criteria (CRS and culture), the results were treated as two separate studies. Using this principle, 7 studies were included with CRS as the gold standard and 6 with culture as the gold standard (Table 1). A total of 502 patients were included in studies in which CRS was considered the gold standard, and a total of 620 patients were included in studies in which culture was considered the gold standard.

**Table 1:** Characteristics of the included studies.

Author	Year	County	N	TP	FP	FN	TN	Reference	Patient population	Income	Sample type	Decontaminate method	Sample condition	Homogenisation	SR	CRS definition	Culture method
Pandie, S.	2014	South Africa	95	44	0	25	26	CRS	Suspected TBP	Low	Pericardial fluid	No	Frozen	Mechanical	-	Culture, histopathology, PF characteristics, and response to treatment	liquid
Sharma, S. K.a	2014	India	20	1	1	3	15	Culture	Suspected TBP	Low	Pericardial fluid	-	-	-	-	-	Solid and liquid
Sharma, S. K.b	2014	India	20	1	0	3	16	CRS	Suspected TBP	Low	Pericardial fluid	-	-	-	-	Smear, culture, histology, PF characteristics and response to treatment	Solid and liquid
Saeed, M.	2017	Pakistan	128	13	0	5	110	Culture	Suspected TBP	Low	Pericardial fluid	-	-	-	-	-	Solid
Ullah, I.	2017	Pakistan	16	4	0	0	12	Culture	Suspected TBP	Low	Pericardial fluid	NALC-NaOH	Fresh	No	3:1	-	Solid and liquid
Yu, G.	2017	China	27	12	0	5	10	CRS	Suspected TBP	Low	Pericardial tissue	NALC-NaOH	Fresh	Mechanical	-	Smear, culture, histology, PF characteristics and response to treatment	Solid and liquid
Khan, A. S.	2018	Pakistan	56	6	0	1	49	CRS	Suspected TBP	Low	Pericardial fluid	NALC-NaOH	Fresh	Mechanical	-	Clinical presentation, radiological finding, culture, and histology	Liquid
Song, J. Q.	2018	China	158	30	0	3	125	Culture	Suspected TBP	Low	Pericardial fluid	NALC-NaOH	Fresh	No	2:1	-	Solid
Allahyartorkaman, M.	2019	Iran	118	2	1	3	112	Culture	Suspected TBP	Low	Pericardial tissue	NALC-NaOH	Fresh	No	2:1	-	Solid
Hu, X.a	2019	China	180	11	24	3	142	Culture	Suspected TBP	Low	Pericardial fluid	NALC-NaOH	Frozen	Mechanical	-	-	Liquid
Hu, X.b	2019	China	180	34	1	18	127	CRS	Suspected TBP	Low	Pericardial fluid	NALC-NaOH	Frozen	Mechanical	-	Culture, histopathology, PF characteristics, and response to treatment	Liquid
Yu, G.	2020	China	19	9	0	5	5	CRS	Suspected TBP	Low	Pericardial fluid and tissue	NALC-NaOH	Fresh	Mechanical	-	Smear, culture, histology, PF characteristics and response to treatment	Solid and liquid
Hu,X.	2020	China	105	26	1	13	65	CRS	Suspected TBP	Low	Pericardial fluid	NALC-NaOH	Fresh	-	-	Culture, histopathology, PF characteristics, and response to treatment	Liquid

CRS, composite reference standard; TBP, tuberculous pericarditis; PF; pericardial fluid; TP, true-positive; FP, false-positive; FN, false-negative; TN, true-negative; NALC-NaOH, N-Acetyl-L-cysteine sodium hydroxide; SR, sample ratio.

### 3.2 Study quality

**Fig 2:**
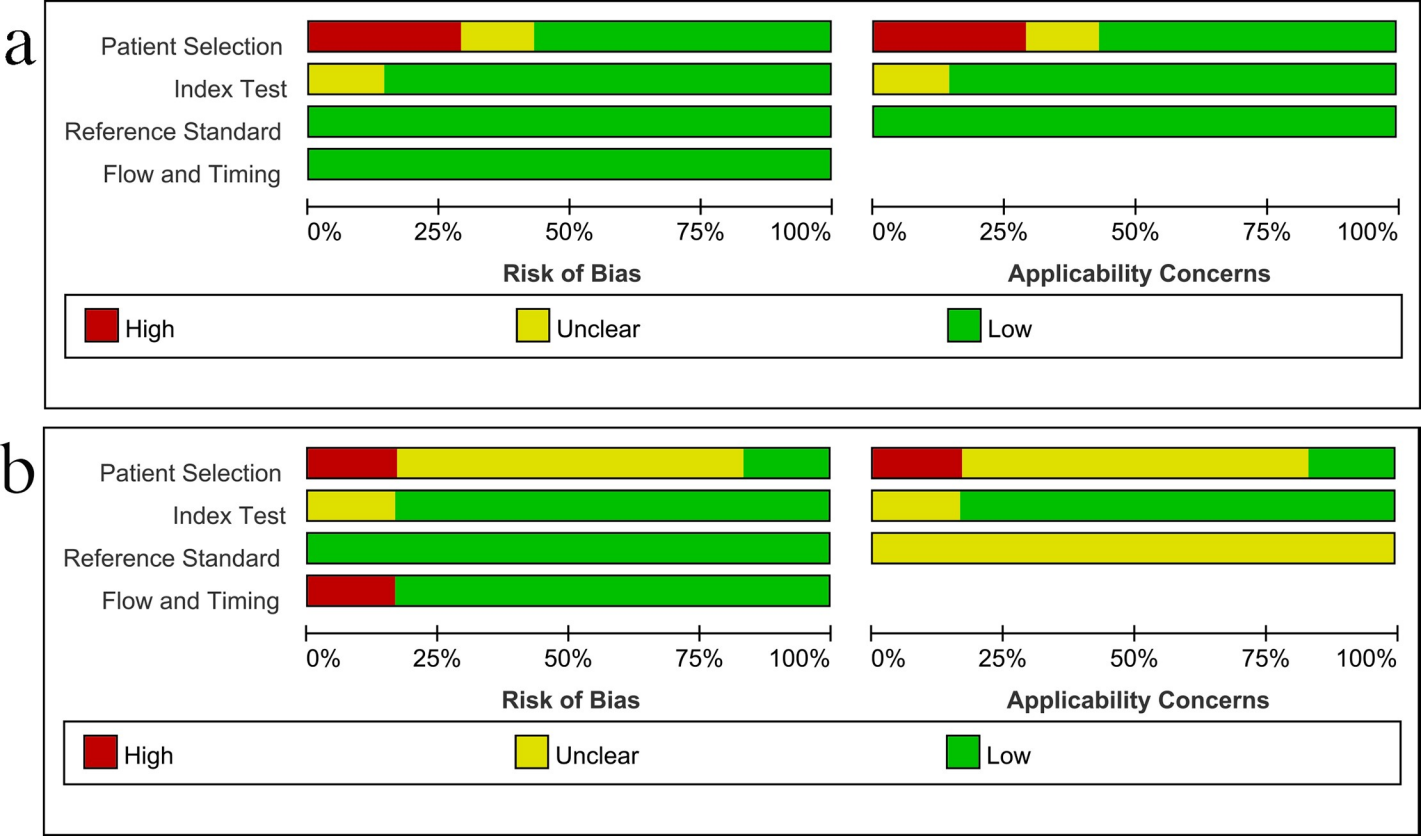
Methodological quality graphs (risk of bias and applicability concerns) as percentages across the included studies. (a) Composite reference standard. (b) Culture reference standard.

Fig 2 shows the results of the methodological quality assessment of the included studies using CRS and culture as gold standards, respectively. Major sources of bias included the method of patient selection and the reference standard used. The flow and timing of the risk of bias from the index test was judged to be relatively low. Publication bias was not performed because there was no appropriate test with adequate statistical power to reliably assess publication bias in the context of diagnostic test accuracy systematic reviews [16, 39].

### 3.3. Diagnostic accuracy of Xpert MTB/RIF assay for TBP

**Fig 3:**
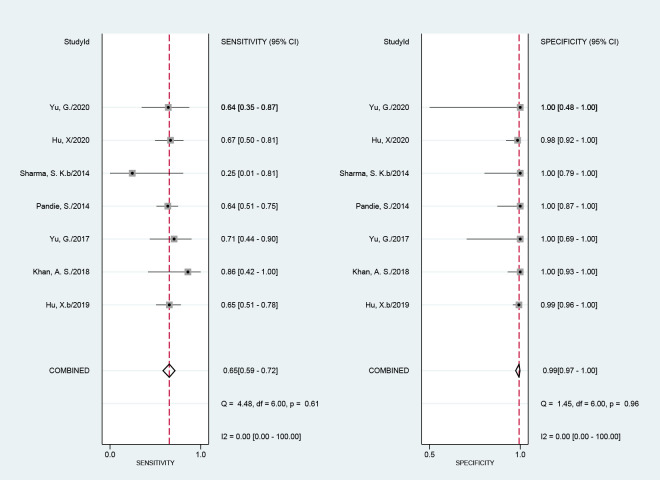
Forest plot of Xpert MTB/RIF sensitivity and specificity for tuberculous pericarditis compared with a composite reference standard.

Seven studies including 502 patients assessed the diagnostic efficacy of Xpert MTB/RIF for TBP using a gold standard of CRS. The sensitivity of Xpert MTB/RIF ranged from 25% (1–81%) to 86% (42–100%). The pooled sensitivity of Xpert MTB/RIF for diagnosis of TBP was 65% (59–72%), with I^2^ = 0%. The specificity ranged from 99% (96–100%) to 100% (93–100%). The pooled specificity of Xpert MTB/RIF for TBP was 99% (97–100%), with I^2^ = 0% (Fig 3). There was no significant heterogeneity of either sensitivity or specificity. The AUC of the SROC was 0.99 (0.97–0.99).

**Fig 4:**
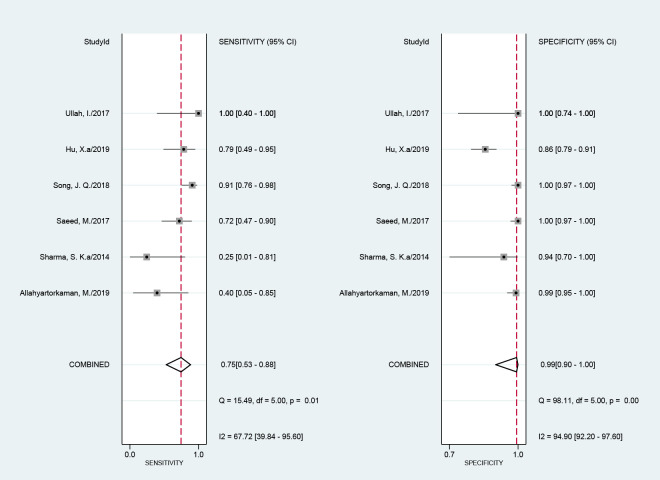
Forest plot of Xpert MTB/RIF sensitivity and specificity for tuberculous pericarditis compared with culture.

Six studies with 620 samples used culture as the reference standard. The Xpert MTB/RIF sensitivity ranged from 25% (1–81%) to 100% (40–100%). The pooled sensitivity of Xpert MTB/RIF was 75% (53–88%), with I^2^ = 68%. The specificity of Xpert MTB/RIF ranged from 86% (79–91%) to 100% (97–100%). The pooled specificity of Xpert MTB/RIF was 99% (90–100%), with I^2^ = 95% (Fig 4). There was substantial heterogeneity of sensitivity and specificity. The AUC of the SROC was 0.94 (95% CI, 0.92–0.96) as compared with culture.

### 3.4 Subgroup and meta-regression analyses

**Fig 5:**
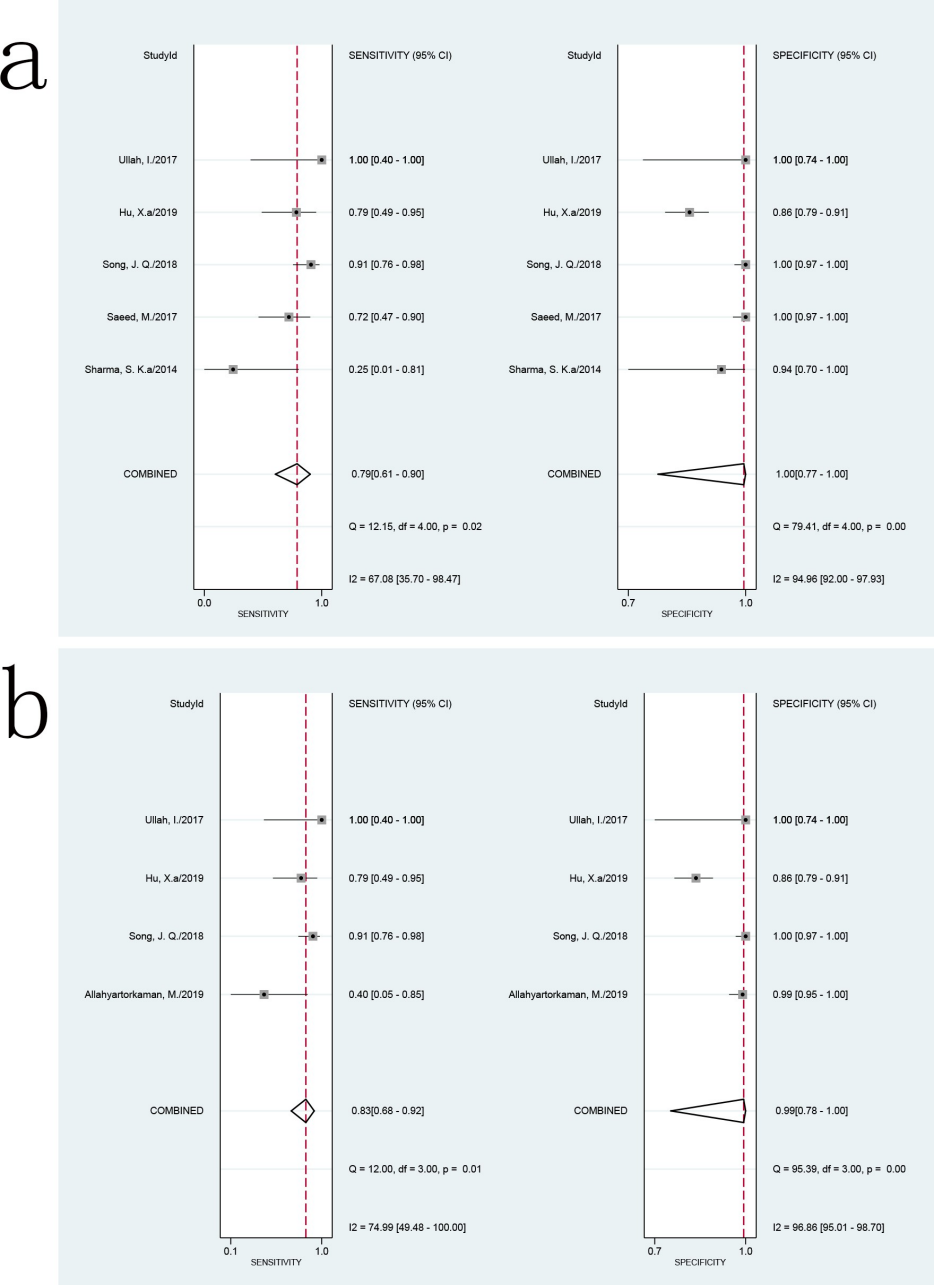
Forest plot of Xpert MTB/RIF sensitivity and specificity for tuberculous pericarditis compared with culture for subgroup analysis. (a) Using pericardial effusion. (b) Using NALC-NaOH for decontamination.

The preliminary analyses showed that the heterogeneity between studies was significant when compared with culture. We explored the heterogeneity among studies using subgroup and meta-regression analyses on predefined subgroups of patient selection methods, sample types, sample conditions, homogenization methods, and decontamination methods used in the assay. In some studies the specific process of specimen processing was not reported; thus, these studies were excluded from the relevant subgroup and meta-regression analyses. When compared with culture, the sensitivity of Xpert MTB/RIF using pericardial effusion ranged from 25% (1–81%) to 100% (40–100%), and the specificity ranged from 86% (79–91%) to 100% (97–100%). The pooled sensitivity and specificity of Xpert MTB/RIF assay using pericardial effusion samples versus culture were 79% (I^2^ = 67%; 61–90%) and 100% (I^2^ = 95%; 77–100%), respectively (Fig 5A). There was a substantial level of heterogeneity in the sensitivity and specificity among studies of Xpert MTB/RIF using pericardial effusion samples compared with culture. The AUC of the SROC was 0.93 (0.90–0.95). When using N-acetyl-L-cysteine-sodium hydroxide (NALC-NaOH) for decontamination, the pooled sensitivity and specificity of Xpert MTB/RIF assay versus culture were 83% (I^2^ = 75%; 68–92%) and 99% (I^2^ = 97%; 78–100%), respectively (Fig 5B). There was a substantial level of heterogeneity among studies. The AUC of the SROC was 0.92 (0.89–0.94). These results suggested that these two factors may not be sources of heterogeneity. Studies related to other parameters (such as homogenization methods, sample conditions) were limited, and we did not perform subgroup analysis.

Meta-regression should generally not be considered when there are fewer than ten studies in a meta-analysis. Therefore, in this study, we did not perform meta-regression analysis. Sensitivity analysis did not identify articles that might be the source of heterogeneity in sensitivity and specificity.

## 4. Discussion

TBP accounts for roughly 1–8% of all new cases of TB [40]. Similar to EPTB, TBP is also paucibacillary, which makes early diagnosis more difficult [41]. A delay in diagnosis or misdiagnosis leads to increased adverse outcomes of TBP [7]. Conventional AFB tests do not have a high enough diagnostic performance in TBP to allow for early and rapid diagnosis. Therefore, to reduce the occurrence of serious adverse reactions to TBP, an early, rapid, and direct method of detecting MTB is urgently needed.

Nucleic acid amplification tests (NAATs), which directly detect nucleic acids of pathogenic bacteria, are widely used for the detection of pathogenic bacteria, such as TB [42]. NAATs are increasingly valued for their efficiency and accuracy of detection, and they play an increasingly important role in the early diagnosis of infectious diseases [43]. Xpert MTB/RIF is currently the most commonly used NAAT for the diagnosis of TB. Xpert MTB/RIF detects MTB DNA via automated half-nest real-time polymerase chain reaction and reports results within two hours [44]. Xpert MTB/RIF has excellent diagnostic efficacy for both PTB and EPTB. The World Health Organization also recommends the use of Xpert MTB/RIF for the early diagnosis of EPTB, including tuberculous meningitis and lymph node tuberculous [45]. This test is also used in the early diagnosis of TBP. Although many relevant studies have reported the diagnostic efficacy of Xpert MTB/RIF in TBP [23, 46, 47], the results were variable. A similar meta-analysis of Xpert for the diagnosis of TBP had been published [48]. However, this study had many shortcomings, such as only assessing the results of comparing with culture and not exploring heterogeneity [48]. Thus, we designed this systematic review and meta-analysis to improve the evaluation of the diagnostic efficacy of the test in TBP.

The present study included seven studies that evaluated Xpert MTB/RIF against a CRS and six studies that evaluated the test against culture. The pooled sensitivity and specificity of Xpert MTB/RIF for TBP were 65% (I^2^ = 0%; 59–72%) and 99% (I^2^ = 0%; 97–100%), respectively, as compared with CRS. There was no observed heterogeneity between the included studies; therefore, we did not perform meta-regression analysis, subgroup analysis, or sensitivity analysis to detect heterogeneity. When using CRS as the gold standard, the results of Xpert MTB/RIF for the diagnostic validity of TBP were highly reliable, with an AUC of the SROC of 0.99. These results suggest that Xpert MTB/RIF had very high diagnostic efficacy for the early diagnosis of TBP. However, when culture was used as the gold standard, the heterogeneity between the included studies was remarkable. We explored the potential sources of heterogeneity using the parameters that were set in advance. Subgroup analysis revealed that the pooled sensitivity and specificity of the Xpert MTB/RIF assay using pericardial effusion samples compared with culture were 79% (I^2^ = 67%; 61–90%) and 100% (I^2^ = 95%; 77–100%), respectively, and the pooled sensitivity and specificity of Xpert MTB/RIF assay using NALC-NaOH for decontamination versus culture were 83% (I^2^ = 75%; 68–92%) and 99% (I^2^ = 97%; 78–100%), respectively. The level of heterogeneity within subgroups was still very significant, which suggested that sample types and decontamination methods might not be a source of heterogeneity for sensitivity and specificity among studies. Two studies did not report specific specimen-processing procedures, and only one of the studies included in this study reported HIV infection status, while the other studies did not report HIV infection status, so we could not perform subgroup analysis for these parameters (sample conditions, HIV infection status and homogenization). For the time being, we cannot evaluate whether these factors are heterogeneous sources of sensitivity and specificity, and more studies are needed to evaluate this issue. Sensitivity analysis did not identify articles that might be the source of heterogeneity. However, in any case, the heterogeneity in sensitivity and specificity was significant, and thus, when culture was used as the gold standard, the relevant results must still be treated with caution. The studies also used different culture references. One study only used BACTEC MGIT 960 liquid culture as the reference, three studies only used the Lowenstein–Jensen solid culture as the reference, and two studies used both as references. The diagnostic efficiency of liquid culture was different from that of solid culture, which might be a source of heterogeneity among studies. Moreover, the number of relevant individual studies in each subgroup was limited, and further analysis could not be performed. The number of studies compared with culture was still relatively small, which might be related to the low culture positivity rate of MTB in pericardium. In addition, culture was not a perfect reference standard; therefore, most of the studies used CRS and included several evaluation factors as a reference standard. CRS might be a source of clinical heterogeneity, but in the present study, the correlation analysis did not suggest significant heterogeneity when CRS was used as the reference standard, and therefore was not discussed further.

This study had several limitations. This was not a meta-analysis based on individual data. Although we searched the relevant literature as comprehensively as possible, some literature might still have been missed. Some studies were unable to distinguish between specific specimen types. In addition, the number of studies that included a comparison with culture was limited and did not allow for further analysis. Moreover, when culture was used as the gold standard, the heterogeneity between studies was significant, and the obtained results should be treated with caution. Finally, this study only included studies in TB endemic areas, no studies in non-TB endemic areas. The role of Xpert MTB/RIF in the diagnosis of TBP may be different in TB endemic and non-endemic areas, because in non-TB endemic areas the likelihood of negative results is much greater, whereas a positive result is of greater concern. In non-TB endemic areas, the incidence of TBP is very low and the role of Xpert MTB/RIF in TBP still needs to be further explored.

According to the GRADE guideline, the evidence quality of this study was high, and the recommendation level was strong when using CRS as the gold standard. When the culture was the gold standard, the quality of evidence was low and the level of recommendation was weak.

## 5. Conclusions

We observed a pooled sensitivity, specificity, and AUC of 65%, 99%, and 0.99, respectively, for the use of Xpert MTB/RIF in the diagnosis of TBP as compared with a CRS, and we found no significant heterogeneity between studies. When Xpert MTB/RIF was compared with culture, the pooled sensitivity, specificity, and AUC of Xpert MTB/RIF were 75%, 99%, and 0.94, respectively, but the heterogeneity was obvious. The associated results needed to be treated with caution when compared with culture. The sensitivity of Xpert MTB/RIF for diagnosing TBP was moderate and the specificity was good; thus, Xpert MTB/RIF can be used in the initial diagnosis of TBP.

## Supporting information

S1 Checklist Data analyzed in this study, including search strategy. PRISMA checklist: PRISMA checklist.
(DOC)

S1 File Search strategy. (DOCX)

S2 File Detailed review of each domain of risk of bias and applicability concerns for each included study. (DOCX)
