# Supplementary material for: Diagnostic accuracy of the Xpert MTB/RIF assay for tuberculous pericarditis: A systematic review and meta-analysis
Source: PLoS One. 2021 Sep 10;16(9):e0257220. doi: 10.1371/journal.pone.0257220 (PMC8432788; doi:10.1371/journal.pone.0257220)
Supplement: S1 File — (DOCX) [file pone.0257220.s002.docx]

Pubmed

#1 “Pericarditis, Tuberculous”[Mesh] OR “Pericarditides, Tuberculous” OR “Tuberculous Pericarditides” OR “Tuberculous Pericarditis”

#2 "Tuberculosis"[Mesh] OR tuberculosis OR Tuberculoses OR “Kochs Disease” OR “Koch's Disease” OR “Koch Disease” OR “Mycobacterium tuberculosis Infection” OR “Infection, Mycobacterium tuberculosis” OR “Infections, Mycobacterium tuberculosis” OR “Mycobacterium tuberculosis Infections”

#3 "Pericardial Effusion"[Mesh] OR “Effusion, Pericardial” OR “Effusions, Pericardial” OR “Pericardial Effusions” OR Hemopericardium OR Chylopericardium OR Chylopericardiums

#4 #2 AND #3

#5 "Extra pulmonary tuberculosis" OR " Extrapulmonary tuberculosis"

#6 #1 OR #4 OR #5

#7 Xpert OR geneXpert

#8 #6 AND #7

Embase

#1 'tuberculous pericarditis'/exp OR 'Pericarditides, Tuberculous' OR 'Tuberculous Pericarditides' OR 'Tuberculous Pericarditis'

#2 'tuberculosis'/exp OR tuberculosis OR Tuberculoses OR 'Kochs Disease' OR 'Koch's Disease' OR 'Koch Disease' OR 'Mycobacterium tuberculosis Infection' OR 'Infection, Mycobacterium tuberculosis' OR 'Infections, Mycobacterium tuberculosis' OR 'Mycobacterium tuberculosis Infections'

#3 'pericardial effusion'/exp OR 'Effusion, Pericardial' OR 'Effusions, Pericardial' OR 'Pericardial Effusions' OR Hemopericardium OR Chylopericardium OR Chylopericardiums

#4 #2 AND #3

#5 'extrapulmonary tuberculosis'/exp OR 'extra pulmonary tuberculosis' OR 'extrapulmonary tuberculosis'

#6 #1 OR #4 OR #5

#7 'xpert'/exp OR xpert OR genexpert

#8 #6 AND #7

Cochrane

#1 “Pericarditis, Tuberculous”[Mesh] OR “Pericarditides, Tuberculous” OR “Tuberculous Pericarditides” OR “Tuberculous Pericarditis”

#2 "Tuberculosis"[Mesh] OR Tuberculoses OR “Kochs Disease” OR “Koch's Disease” OR “Koch Disease” OR “Mycobacterium tuberculosis Infection” OR “Infection, Mycobacterium tuberculosis” OR “Infections, Mycobacterium tuberculosis” OR “Mycobacterium tuberculosis Infections”

#3 "Pericardial Effusion"[Mesh] OR “Effusion, Pericardial” OR “Effusions, Pericardial” OR “Pericardial Effusions” OR Hemopericardium OR Chylopericardium OR Chylopericardiums

#4 #2 AND #3

#5 "Extra pulmonary tuberculosis" OR " Extrapulmonary tuberculosis"

#6 #1 OR #4 OR #5

#7 Xpert OR geneXpert

#8 #6 AND #7

Wanfang and CNKI

#1 (结核 AND 心包) OR 肺外结核

#2 xpert OR genexpert

#3 #1 and #2
