# Supplementary material for: Diagnostic accuracy of the Xpert MTB/RIF assay for tuberculous pericarditis: A systematic review and meta-analysis
Source: PLoS One. 2021 Sep 10;16(9):e0257220. doi: 10.1371/journal.pone.0257220 (PMC8432788; doi:10.1371/journal.pone.0257220)
Supplement: S2 File — (DOCX) [file pone.0257220.s003.docx]

Detailed review of each domain of risk of bias and applicability concerns for each included study.

CRS (1. Hu, X. 2020; 2. Yu, G. 2020; 3. Hu, X.b 2019; 4. Khan, A. S. 2017; 5. Yu, G. 2017; 6. Pandie, S. 2014; 7. Sharma, S. K.b 2014;)

1. Was a consecutive or random sample of patients: all patients defined for inclusion included consecutively Yes (1,3,5,6 consecutive) No (2,4 convenience) Unclear (7 unreported)
2. Was a case-control design avoided? Yes (1-7)
3. Did the study avoid inappropriate exclusions? Were all samples collected analyzed? Yes (1-7)
   - Patient selection risk of bias: High risk (2,4 convenience); low risk (1,3,5,6 consecutive); unclear risk of bias (7 unreported)
   - Patient applicability: High risk (2,4 convenience); low risk (1,3,5,6 consecutive); unclear risk of bias (7 unreported)
4. Were the index test results interpreted without knowledge of the results of the reference standard? Yes (1-7)
5. If a threshold was used (for index test), was it pre-specified? Yes (1-6) Unclear (7 unreported)
   - Index test risk of bias: low risk (1-6); unclear risk of bias (7 unreported).
   - Index test applicability: High risk; low risk, unclear risk.
6. Is the reference standard likely to correctly classify the target condition? Yes (1-7)
7. Were the reference standard results interpreted without knowledge of the results of the index test? Yes (1-7)
   - Reference standard risk of bias: Low risk (1-7)
   - Reference standard applicability: low risk (1-7)
8. Was there an appropriate interval between index test(s) and reference standard? Yes (1-7)
9. Did all patients receive a reference standard? 100% of samples analyzed for the index test received the reference standard Yes (1-7)
10. Did patients receive the same reference standard? Yes (1-7)
11. Were all patients included in the analysis? Yes (1-7)
    - Flow and time risk of bias: low risk (1-7)

Culture (1. Allahyartorkaman, M. 2019; 2. Hu, X.a 2019; 3. Song, J. Q. 2018; 4. Saeed, M. 2017; 5. Ullah, I. 2017; 6. Sharma, S. K.a 2014)

1.Was a consecutive or random sample of patients: all patients defined for inclusion included consecutively Yes (2) No(1, convenience) Unclear(3,4,5,6 unreported)

2. Was a case-control design avoided? Yes (1-6)

3. Did the study avoid inappropriate exclusions? Were all samples collected analyzed? Yes(1,2,3,4,6) Unclear(5, not all samples)

Patient selection risk of bias: High risk (1, convenience); low risk (2); unclear risk of bias (3,4,5,6 unreported)

- - Patient applicability: High risk (1, convenience); low risk (2); unclear risk of bias (3,4,5,6 unreported)

4. Were the index test results interpreted without knowledge of the results of the reference standard? Yes (1-6)

5. If a threshold was used (for index test), was it pre-specified? Yes (1-6)

- - Index test risk of bias: low risk (1-6).
  - Index test applicability: low risk (1-6)

6. Is the reference standard likely to correctly classify the target condition? Unclear (1-6, Pericardial effusion MTB culture positivity rate is low, therefore, the classification for disease status may be imperfect)

7. Were the reference standard results interpreted without knowledge of the results of the index test? Yes (1-6)

- - Reference standard risk of bias: low risk (1-6)
  - Reference standard applicability: unclear risk of bias (1-6)

8. Was there an appropriate interval between index test(s) and reference standard? Yes (1-6)

9. Did all patients receive a reference standard? 100% of samples analyzed for the index test received the reference standard Yes (1-6)

10. Did patients receive the same reference standard? Yes (1-6)

11. Were all patients included in the analysis? Yes (2-6) No (1, not all patients)

- - Flow and time risk of bias: High risk (1); low risk (2-6)
